# ING2 Controls Mitochondrial Respiration *via* Modulating MRPL12 Ubiquitination in Renal Tubular Epithelial Cells

**DOI:** 10.3389/fcell.2021.700195

**Published:** 2021-08-09

**Authors:** Ying Yang, Chensheng Li, Xia Gu, Junhui Zhen, Suwei Zhu, Tingting Lv, Qiang Wan, Yi Liu

**Affiliations:** ^1^Department of Pharmacy, The Affiliated Hospital of Shandong University of Traditional Chinese Medicine, Jinan, China; ^2^Department of Endocrinology, Shandong Provincial Hospital Affiliated to Shandong First Medical University, Jinan, China; ^3^Department of Gastrointestinal Surgery, Shandong Provincial Hospital, Cheeloo College of Medicine, Shandong University, Jinan, China; ^4^School of Medicine, Cheeloo College of Medicine, Shandong University, Jinan, China; ^5^Department of Pathology, Cheeloo College of Medicine, Shandong University, Jinan, China; ^6^Central Laboratory, Jinan Central Hospital Affiliated to Shandong University, Jinan, China; ^7^Department of Pulmonary and Critical Care Medicine, Shandong Provincial Hospital, Cheeloo College of Medicine, Shandong University, Jinan, China; ^8^Department of Pulmonary and Critical Care Medicine, Shandong Provincial Hospital Affiliated to Shandong First Medical University, Jinan, China; ^9^Shandong Key Laboratory of Infectious Respiratory Disease, Jinan, China

**Keywords:** ING2, MRPL12, mitochondrial respiration, ubiquitination, tubular epithelial cells, kidney disease

## Abstract

Mitochondrial injury of tubular epithelial cells (TECs) is the key pathogenic event underlying various kidney diseases and a potential intervening target as well. Our previous study demonstrated that ING2 is ubiquitously expressed at tubulointerstitial area within kidneys, while its role in regulating TEC mitochondrial respiration is not fully elucidated. To clarify the roles of ING2 in mitochondrial homeostasis of TECs and pathogenesis of acute ischemic kidney injury, Western blot, PCR, immunofluorescence, immunoprecipitation, and oxygen consumption rate assay were applied to address the roles of ING2 in modulating mitochondrial respiration. We further complemented these studies with acute ischemic kidney injury both *in vitro* and *in vivo*. *In vitro* study demonstrated ING2 could positively control TEC mitochondrial respiration. Concurrently, both mRNA and protein levels of mtDNA encoded respiratory chain components were altered by ING2, suggesting ING2 could regulate mtDNA transcription. In mechanism, ING2 could regulate the ubiquitination of a newly identified mitochondrial transcription factor MRPL12, thereby modulating its cellular stability and abundance. We also demonstrated ING2-mediated modulation on mtDNA transcription and mitochondrial respiration are involved in serum deprivation induced TEC injuries. Finally, immunohistochemistry study revealed that ING2 expression was significantly altered in kidney biopsies with acute ischemic kidney injury. *In vivo* study suggested that kidney specific ING2 overexpression could effectively ameliorate acute ischemic kidney injury. Our study demonstrated that ING2 is a crucial modulator of TEC mitochondrial respiration. These findings suggested a unrecognized role of ING2 in TEC mitochondrial energetic homeostasis and a potential intervening target for TEC mitochondrial injury associated pathologies.

## Introduction

Due to their heavy tasks in maintaining fluid and electrolyte balance *via* reabsorption and excreting, kidney tubular epithelial cells (TECs) are one of the most highly energy-demanding cell types within the body and mainly rely their energy supply on mitochondrial oxidative phosphorylation (OXPHOS) (Govers et al., 2020). In this sense, mitochondrial activity, as well as its fine-tuned adaptions to environmental challenges, is essential for TEC biology. The importance of mitochondria in TECs is clearly highlighted by the manifestations of several inherited mitochondrial disorders, such as Kearns-Sayre syndrome (Emma et al., 2006) and MELAS syndrome (Seidowsky et al., 2013), which often exhibit obvious renal tubular injuries. Furthermore, the highly energetic reliance of TECs on mitochondria also makes them one of the most susceptible cell types to ischemia or hypoxic insults, presented by a clinic process termed as acute kidney injury (AKI) (Brooks et al., 2009; Funk and Schnellmann, 2012). Besides, recent studies also revealed mitochondrial dysfunction might also participate in the development of chronic kidney diseases (CKD) such as diabetic nephropathy (Galvan et al., 2019; Li et al., 2020). To the current notion, mitochondrial dysfunction caused by hyperglycemia might not only worsen the already-existed cellular metabolic disturbance but also provoke oxidative stresses, accordingly leading to caspase activation and cell apoptosis (Dugan et al., 2013; Zhang et al., 2018; Sun et al., 2019). Thus, mitochondria have emerged as one common therapeutic target for several lines of kidney disorders, including both inherited and acquired ones (Emma et al., 2016; Sun et al., 2019). However, as no effective treatments aiming at improving mitochondrial quality are available yet, a better understanding of mitochondrial biology of TECs, along with its regulatory mechanisms, requires further exploration.

ING2, a member of inhibitor of growth (ING) family, is best known as a tumor suppressor for its expression is often lost or reduced in several tumors, such as non-small cell lung carcinoma (Ythier et al., 2010), head and neck squamous cell carcinoma (Borkosky et al., 2009), and hepatocellular carcinoma (Zhang et al., 2008). Functional studies revealed ING2 could modulate ample cellular biological processes including cell cycle, DNA repair, apoptosis, senescence, and chromatin remodeling (Guerillon et al., 2013; Archambeau et al., 2019). The major mechanism mediating ING2’s effects above is to deacetylating histone *via* interacting with mSin3a/HDAC1-2/Sap30 complex, thereby modifying target gene expression (Shi et al., 2006). In addition, ING2 was also evidenced to control p53 signaling. Currently, ING2 has emerged as one potential intervening target for cancers (Nagashima et al., 2001; Pedeux et al., 2005). However, the role of ING2 in mitochondrial function and renal biology is largely unknown.

In the present study, we identified ING2 was also involved in the maintenance of renal TEC homeostasis by regulating mitochondrial respiration. First, ING2 is a positive regulator of TEC mitochondrial DNA (mtDNA) transcription and respiration. In mechanism, the effects of ING2 on mtDNA transcription were mediated by MRPL12 (mitochondrial ribosomal protein L12), a recently identified mitochondrial transcription regulator. ING2 could modulate the ubiquitination of MRPL12, thereby controlling its cellular stability and abundance. Second, ING2 mediated modulation on mtDNA transcription and mitochondrial respiration are involved in serum deprivation induced TEC injury. Finally, ING2 expression was significantly altered in kidney biopsies of AKI, and kidney specific ING2 overexpression could effectively ameliorate ischemic AKI mouse models. These findings suggested a novel role of ING2 in TEC mitochondrial energetic homeostasis and a potential intervening target for TEC mitochondrial injury associated pathologies.

## Materials and Methods

### Animals

C57BL/6J male mice, aged 8–10 weeks, were purchased from Jinan Pengyue Experimental Animal Breeding Co. Ltd. All mice were maintained in the animal facilities under specific pathogen-free conditions with free access to food and water. All the experimental protocols were approved by the institutional ethics committee.

### Cell Culture

HK2 cells (ATCC, CRL-2190) were cultured in Dulbecco’s modified Eagle’s medium (DMEM) containing 10% fetal bovine serum (FBS), 100 units/ml penicillin, and 100 mg/ml streptomycin at 37°C in a 5% CO_2_ incubator. For serum deprivation experiments, HK2 cells were cultured in DMEM containing 100 units/ml penicillin and 100 mg/ml streptomycin for 48 h.

### Transfection

Small interfering RNA (siRNA) targeting ING2 and non-targeting negative control siRNA were purchased from BioSune (China). HK2 cells were transfected with 50 nM siRNA by using Lipofectamine 3000 reagent (Invitrogen) for 48 h. For ING2 over-expression assay, cells were transfected ING2 plasmid (pcDNA3.1-ING2, BioSune) or control plasmid (pcDNA3.1, BioSune) using Lipofectamine 3000 reagent (Invitrogen) for 48 h. For co-transfection assay, HK2 cells were treated with MRPL12 knock-out lentiviruses (BioSune) or control lentiviruses (BioSune), following transfection with ING2 plasmid or control plasmid 24 h later.

### Oxygen Consumption Rate

Oxygen consumption rate (OCR) was assessed using Seahorse XF Cell Mito Stress Test Kit (Agilent Technologies, 103015-100) according to the manufacturer’s instructions. Before the assay, an equal number of cells (10^4^/well) were transferred into Seahorse plate. Also, 2 μM oligomycin, 1.5 μM FCCP [carbonyl cyanide 4-(trifluoromethoxy) phenylhydrazone], 0.5 μM rotenone, and 0.5 μM antimycin were used in the assay.

### Intra-Renal Injection of rAAV Vectors

Control rAAV vector (rAAV-con) and rAAV vectors encoding the human ING2 genes (rAAV-ING2) were produced from BioSune (China). For intra-renal injection of rAAV vectors, mice were anesthetized with an intraperitoneal injection of sodium pentobarbital, and the bilateral kidneys were exposed *via* a flank incision. rAAV vectors (2 × 10^9^ genome copies/mouse) were slowly injected into the kidney using a 29-G needle.

### Animal Model of Kidney Ischemia Reperfusion Injury

Mice were anesthetized by intra-peritoneal injection with sodium pentobarbital, after which the bilateral kidneys were exposed through flank incisions. The bilateral renal pedicle was clamped using a straight clip for 45 min and then released. Animals were then allowed to recover, with free access to food and water. Forty-eight hours later, the mice were sacrificed, and the kidneys, blood, and urine were collected for further analysis.

### Detection of Creatinine in Serum and Urine

Mice were sacrificed 48 h after the induction of ischemia reperfusion injury. The blood and urine were collected. The serum was isolated by centrifuged at 3,000 *g* for 10 min. The levels of creatinine in serum and urine were detected using Creatinine (Cr) Assay kit (sarcosine oxidase, Nanjing Jiancheng Bioengineering Institute) according to the manufacturer’s instruction.

### Western Blot

Cells or kidney tissues were lysed in RIPA buffer (Beyotime, P0013B) containing 1% phenylmethylsulfonyl fluoride (PMSF, Solarbio, P0100). The supernatants were obtained after centrifugation at 12,000 rpm for 15 min. Protein concentrations were determined using BCA Protein Assay Kit (Solarbio, PC0020). Lysates were diluted in 4 × SDS-PAGE loading buffer (Solarbio, P1015) and boiled at 95°C for 5 min. The samples were resolved by SDS-PAGE (Beyotime Biotechnology) and then transferred to PVDF membrane (Millipore). After blocking with 0.5% skimmed milk, the membrane was incubated with rabbit anti-ING2 antibody (Proteintech, 11560-1-AP, 1:1,000), mouse anti-β-actin antibody (Proteintech, 60008-1-Ig, 1:5,000), rabbit anti-ND1 antibody (Proteintech, 19703-1-AP, 1:1,000), rabbit anti-ND2 antibody (Proteintech, 19704-1-AP, 1:1,000), rabbit anti-ND5 antibody (Proteintech, 55410-1-AP, 1:1,000), rabbit anti-ND6 antibody (Absin, Abs139196, 1:1,000), rabbit anti-CYTB antibody (Proteintech, 55090-1-AP, 1:1,000), rabbit anti-COX II antibody (Proteintech, 55070-1-AP, 1:1,000), rabbit anti-COX I antibody (Bioss, bs-3953R, 1:1,000), rabbit anti-ATP6 antibody (Proteintech, 55313-1-AP, 1:1,000), rabbit anti-ATP8 antibody (Proteintech, 26723-1-AP, 1:1,000), rabbit anti-MRPL12 antibody (Proteintech, 14795-1-AP, 1:1,000), rabbit anti-TFAM antibody (Proteintech, 22586-1-AP, 1:1,000), and rabbit anti-ubiquitin antibody (Proteintech, 10201-2-AP, 1:1,000). Then the membrane was incubated with horseradish peroxidase (HRP)-conjugated anti-rabbit or anti-mouse IgG antibody and detected by chemiluminescence reagents (ECL, Millipore).

### qRT-PCR

Total RNA was isolated by using TRIzol solution (Invitrogen, 15596026). cDNA was then synthesized using PrimeScript^TM^ RT reagent kit (Takara, No. RR047A). Quantitative RT-PCR reactions were performed with TB Green^®^ Premix Ex Taq^TM^ II (Takara, No. RR820L). The primer pairs used for PCR analyses were as follows: β-actin, forward TGGCACCCAGCACAATGAA and reverse CTAAGTCATAGTCCGCCTAGAAGCA; ING2, forward GCGAGAGCTGGACAACAAAT and reverse GACAC TTGGTTGCATAAGCAG; MRPL12, forward ATCCAGG ATGTCGGGCTTG and reverse TGATGCCTTGGATGTAGT TCTTGA; ND1, forward CGAGCAGTAGCCCAAACAATC and reverse GATGGCAGGAGTAATCAGAGGTG; ND2, forward ACCATCTTTGCAGGCACACT and reverse GCTTCTGTG GAACGAGGGTT; ND5, forward TCAGTTGATGATACGCCC GA and reverse TGGGGTGAGGCTTGGATTAG; ND6, forward TTGGTGCTGTGGGTGAAAGA and reverse ATCAACC CTGACCCCTCTCC; CYTB, forward CCCACCCCATCCA ACATCTC and reverse GCGTCTGGTGAGTAGTGCAT; COX I, forward CAAACGCCCCTTTTCGTCT and reverse GTGTT GAGGTTGCGGTCTGTT; COX II, forward CATGAGCTG TCCCCACATTAG and reverse CGGTCGTGTAGCGGTGAAA; ATP6, forward ACCACAAGGCACACCTACAC and reverse TATTGCTAGGGTGGCGCTTC. The level of each gene was normalized to the levels of the mouse β-actin gene, and the results were analyzed by the method of quantitative relative expression 2^–ΔΔCT^.

### Immunofluorescence and Immunohistochemistry

To label mitochondria, HK2 cells were washed with pre-warm phosphate buffered saline (PBS) and replenished with warm DMEM containing 100 nM MitoTracker^TM^ Red CMXRos (Invitrogen^TM^, M7512). After incubation for 15 min, cells were washed with PBS for three times for further analysis.

For immunofluorescent staining, cells were fixed with 4% paraformaldehyde, permeabilized in 0.5% Triton X-100, and blocked with 0.5% BSA. Cells were labeled with antibodies against ING2 (Proteintech, 11560-1-AP, 1:200) or MRPL12 (Proteintech, 14795-1-AP, 1:200) at 4°C overnight. Then, cells were stained with the secondary antibody (Alexa Fluor^®^ 488 Goat Anti-Rabbit IgG H&L, ab150077, 1:500) for 1 h, followed by counterstaining with DAPI for 5 min.

For immunohistochemical stain, kidney samples were fixed, paraffin-embedded, and sectioned into 3 μm thick slices. The tissue sections were deparaffinized in dimethylbenzene and rehydrated in gradient alcohol, followed by antigen retrieval in citric acid buffer (pH = 6.0). After cooling to room temperature, sections were washed with PBS and blocked with 10% goat serum for 30 min. Sections were stained with rabbit antibodies against ING2 (1:200, Proteintech), MRPL12 (1:200, Proteintech), ND2 (1:200, Proteintech), or COX II (1:200, Proteintech) overnight at 4°C and incubated with the secondary antibody (HRP labeled goat anti-rabbit IgG, 1:1,000) for 1 h at RT. Finally, slides were colored by DAB staining and hematoxylin re-dyeing. Images were obtained using Leica DMi8 microscope.

### Immunoprecipitation

Immunoprecipitation for MRPL12 was performed with Pierce^TM^ Crosslink IP Kit (Thermo Scientific, 26147) according to the manufacturer’s instructions. In brief, 3 μg of MRPL12 antibody or rabbit control IgG was coupled to 20 μl of Protein A/G Agarose. After treated with proteasome inhibitor MG132 (Sigma, M7449-200UL) for 6 h, cells were lysed with IP Lysis Buffer. Cell lysates were centrifugated at 12,000 rpm for 15 min to remove debris and were used as input. Protein concentrations of lysates were assessed by BCA Protein Assay Kit. Then, 1 mg of cell lysates was incubated with antibody coupling Protein A/G Agarose overnight. The agaroses were washed, and the antigens were eluted with elution buffer. Finally, the samples were analyzed by using Western blot as described above.

### MRPL12 Ubiquitin *in situ* Detection

MRPL12 ubiquitin *in situ* detection was carried out by using Duolink^TM^ *In Situ* PLA kit (Sigma, DUO92101), in combination with mouse anti-MRPL12 antibody and rabbit anti-ubiquitin antibody (Proteintech, 10201-2-AP, 1:200) according to the manufacturer’s instructions.

### Flow Cytometry

Detection of HK2 cell apoptosis was performed using BD Pharmingen^TM^ PE Annexin V Apoptosis Detection Kit I (BD Biosciences, 559763) in accordance with the manufacturer’s instructions. In brief, cells were harvested after trypsin digestion, centrifuged, and washed with Annexin V binding buffer for three times. Then, cells were stained with PE Annexin V and 7-AAD at room temperature for 15 min and analyzed by flow cytometry (BD Aria II). Flow cytometry data were analyzed using FlowJo software (version 10).

### Statistical Analyses

Statistical analysis was performed using the GraphPad Prism 6 software. Data were expressed as the mean ± SEM. Comparisons of the data were performed by Student’s *t*-test and one way ANOVA, and *p* < 0.05 considered to be statistically significant.

## Results

### ING2 Positively Regulates Mitochondrial Respiration in Tubular Epithelial Cells

**FIGURE 1:**
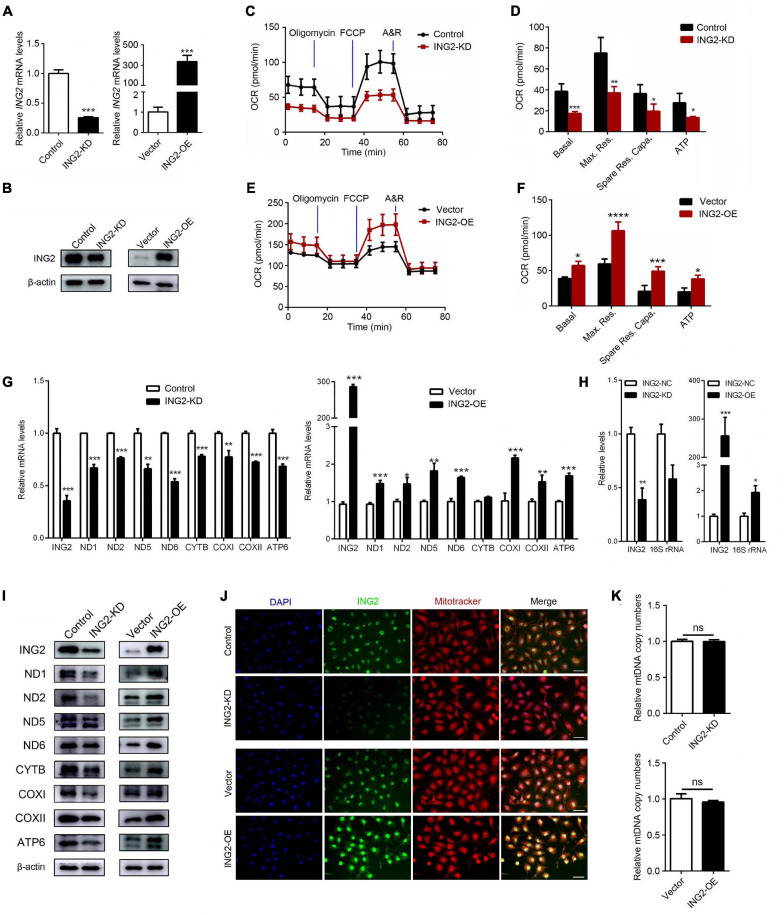
ING2 positively regulates mitochondrial respiration in tubular epithelial cells. HK2 cells were transfected with ING2-shRNA or ING2-overexpression plasmid. **(A,B)** The transfection efficiencies were evaluated by qPCR and Western blotting, respectively. **(C–F)** Following transfection, mitochondrial OXPHOS of HK2 cells were detected using Seahorse. OCR, oxygen consumption rate; FCCP, carbonyl cyanide 4-(trifluoromethoxy)phenylhydrazone; A&R, antimycin and rotenone. **(G,H)** mtDNA-encoded components for complex I, III, IV, and V and 16S rRNA were determined by qPCR. **(I)** mtDNA-encoded components for complex I, III, IV, and V were determined by Western blotting. **(J)** The mitochondria were stained with MitoTracker (red) and ING2 (green) followed by DAPI (blue) re-dyeing. **(K)** The mitochondria DNA (mtDNA) copy number was determined by qPCR with G6PC serving as internal reference. Data were from three individual experiments and presented as mean ± SEM. *p < 0.05; **p < 0.01; ***p < 0.001; ****p < 0.0001. ns, not significant.

Current notion suggested mitochondrial dysfunction serves as one common pathogenic event of ample lines of kidney disorders. The findings that ING2 mainly located at tubulointerstitial area and its expressions were altered under different kidney pathologies raised our interest to observe whether ING2 might have some impacts on TEC mitochondria. First, the effects of ING2 on mitochondrial OXPHOS capability were investigated. In cultured TECs, ING2 was either knocked down or overexpressed and efficiencies were evidenced by qPCR and Western blotting separately (Figures 1A,B). Cell bioenergetic profiles were analyzed by Seahorse XFe96. ING2 knockdown led to a significant decrease of basal OCR, maximal respiration, spare respiratory capacity, and ATP-linked OCR (Figures 1C,D), while ING2 overexpression exerted opposite effects (Figures 1E,F). These results suggested ING2 might act as a positive regulator of mitochondrial OXPHOS. Second, as mitochondrial OXPHOS capability is closely related to the protein expressions of the components within respiratory chain complexes, we continued to examine the effects of ING2 on mtDNA expression. Both mRNA and protein levels of several key mtDNA encoded components consisting of complex I, III, IV, and V were determined separately. Minus or plus cellular ING2 resulted in significant reductions or elevations of protein contents of these mtDNA encoded subunits (Figure 1I), indicating ING2 induced elevation of mitochondrial OXPHOS was mediated by promoting mtDNA expression. Besides, the alterations of mRNA levels of these mtDNA encoded subunits after ING2 minus or plus further evidenced that ING2’s effects might act on mtDNA transcription machinery (Figure 1G). In addition, the effects of ING2 on mtDNA transcription might not be specific for OXPHOS genes, as mitochondrial 16S rRNA, which is essential for mitochondrial ribosome biogenesis, was also modulated by ING2 (Figure 1H). Third, considering that mtDNA transcription is often functionally linked to mitochondrial biogenesis, we also examined the effects of ING2 on mitochondrial abundance. As shown in Figure 1J, MitoTracker staining revealed no changes in mitochondrial abundance in either ING2 minus or plus HK2 cells. The mtDNA copy number analysis further confirmed that ING2 had no effects on mitochondrial biogenesis in HK2 cells (Figure 1K), revealing that the effects of ING2 on OXPHOS are independent of mitochondrial biogenesis.

### ING2 Regulates mtDNA Transcription *via* MRPL12 but Not TFAM

**FIGURE 2:**
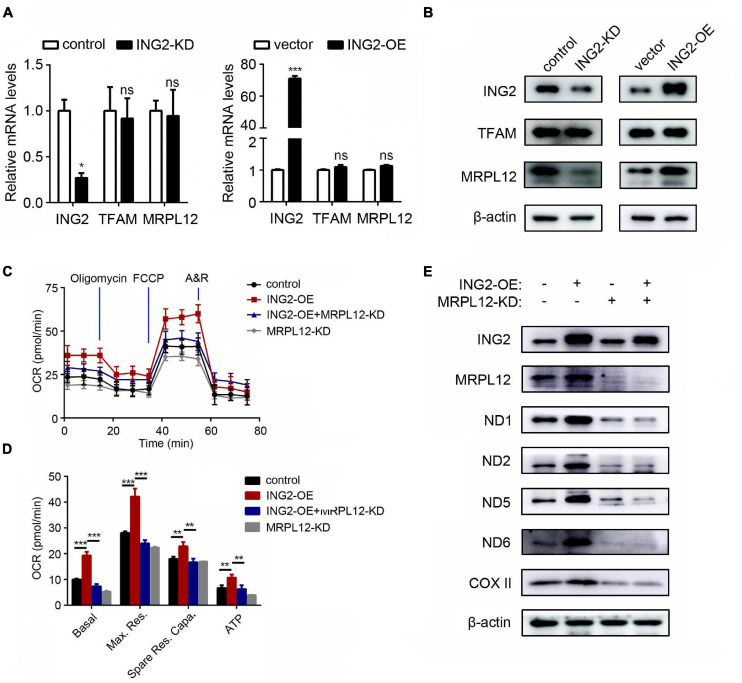
MRPL12 mediated the effects of ING2 on mitochondrial OXPHOS in tubular epithelial cells. **(A,B)** HK2 cells were transfected with ING2-shRNA or ING2-overexpression plasmid. The mRNA and protein levels of TFAM and MRPL12 were evaluated by qPCR and Western blotting. **(C,D)** HK2 cells were divided into control group, ING2 overexpression group, MRPL12 knockout group, and MRPL12 knockout plus ING2 overexpression group. Mitochondrial OXPHOS was detected using Seahorse. OCR, oxygen consumption rate; FCCP, carbonyl cyanide 4-(trifluoromethoxy)phenylhydrazone; A&R, antimycin and rotenone. **(E)** Western blotting to validate the altered expression of mtDNA-encoded components of mitochondria complex. Data were from three individual experiments and presented as mean ± SEM. *p < 0.05, **p < 0.01, ***p < 0.001. ns, not significant.

As ING2 was evidenced capable of inducing mtDNA transcription, we looked into the possible transcriptional regulators of mtDNA. The best elucidated mitochondrial transcriptional factor is TFAM, which has multiple functions on mitochondrial genome such as initiating transcription and replication and maintaining mtDNA stability. However, our results showed that ING2 minus or plus did not alter either mRNA or protein levels of TFAM (Figures 2A,B), suggesting TFAM might not be involved. Then, we investigated the role of MRPL12, a mitochondrial ribosomal protein that was recently identified capable of promoting mtDNA transcription as well. Although mRNA contents of MRPL12 remain unaffected by ING2 minus or plus (Figure 2A), its protein levels significantly decreased or increased correspondingly (Figure 2B). These results suggested MRPL12 might be the possible mediator responsible for ING2’s effects on mtDNA expression. This hypothesis was further confirmed by our following studies. The HK2 cells were dual transfected with ING2 overexpressing and MRPL12 silencing plasmids, and the mitochondrial respiration was measured by seahorse. The elevated basal OCR, maximal respiration, spare respiratory capacity, and ATP-linked OCR induced by ING2 overexpression were significantly reduced by additional MRPL12 suppression (Figures 2C,D). Concurrently, ING2 induced elevation of electron transport chain (ETC) complex expression was also reduced by additional MRPL12 knockdown (Figure 2E). Collectively, these results demonstrated that MRPL12 mediated the effects of ING2 on mtDNA expression and OXPHOS in HK2 cells.

### ING2 Regulated the Ubiquitination of MRPL12

**FIGURE 3:**
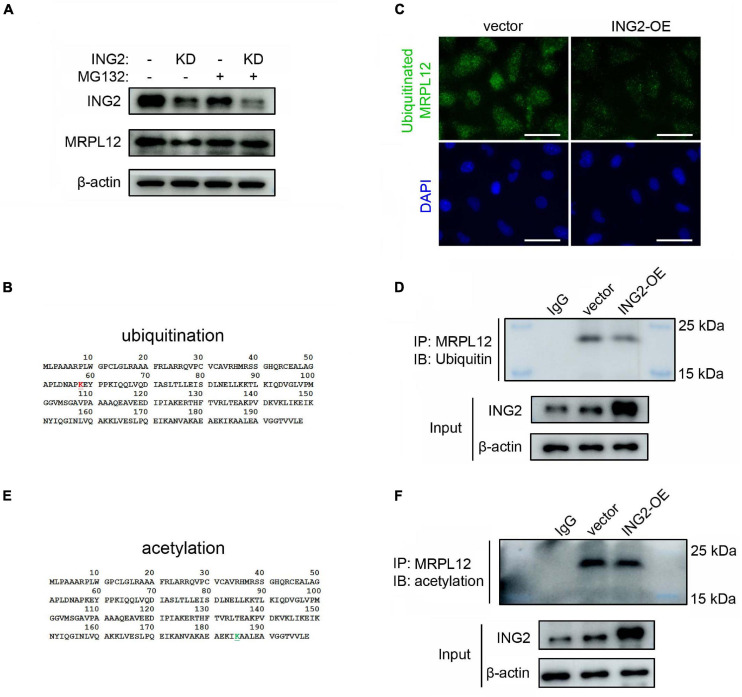
ING2 inhibited the ubiquitination of MRPL12. **(A)** HK2 cells were transfected with control or ING2-shRNA, followed by MG132 treatment for 6 h. Protein levels of ING2 and MRPL12 were evaluated by Western blotting. **(B)** A ubiquitin modified site was found at 58 lysine residue (red, upper panel), predicted using predictor of protein ubiquitination sites, UbPred. **(C,D)** The ubiquitination of MRPL12 was determined by proximity ligation assay (PLA) and immunoprecipitation, respectively, after transfected ING2-overexpression plasmid. **(E)** By using ASEB, a web tool for predicting protein acetylation site, a acetylation site was detected at the 185 lysine residue (green, lower panel). **(F)** The acetylation of MRPL12 was determined by immunoprecipitation after transfected ING2-overexpression plasmid. IP, immunoprecipitation; WB, Western blotting. Data were from three individual experiments.

The above results revealed that ING2 could positively control the protein content of MRPL12 without affecting its mRNA levels, indicating ING2 might have some effects on MRPL12 protein quality control. Such hypothesis was confirmed by the results that the administration of MG132, the inhibitor of proteasomal degradation, could effectively ameliorate ING2 knockdown induced reduction of MRPL12 protein contents (Figure 3A). Ubiquitination is the major degradation manner for cellular proteins, and ING family members have been reported to be capable of modulating the ubiquitination status of other proteins (Thalappilly et al., 2011; Wong et al., 2011). Thereby, we continued to explore if ING2 could regulate the ubiquitination of MRPL12. First, a potential ubiquitin binding site at 58 lysine residue within MRPL12 molecular was determined by using predictor of protein ubiquitination sites UbPred (Figure 3B). Subsequently, the ubiquitinated MRPL12 after ING2 overexpression was measured by proximity ligation assay (PLA), which gives a positive green signal (ubiquitinated MRPL12) when the two proteins of interest (MRPL12 antibody and ubiquitin antibody) are within 30–40 nm. As observed by *in situ* PLA, the ubiquitination of MRPL12 was dramatically reduced in ING2 overexpressed cells compared to the empty vector transfected control cells (Figure 3C). Such findings were further confirmed by our immunoprecipitation experiment that the ubiquitination level of MRPL12 was decreased in ING2 overexpressed HK2 cells (Figure 3D).

As mentioned above, ING2 was reported to exert its biological effects *via* acetylating target proteins. Using Acetylating Set Enrichment Based method (ASEB), we also found there is a potential acetylating site at its 185 lysine residue (Figure 3E). However, our IP experiments showed that the acetylated MRPL12 was not altered by the overexpression of ING2 (Figure 3F), suggesting ING2 did not modulate the acetylation status of MRPL12.

### ING2 Mediated Modulation on Mitochondrial OXPHOS Participates in Ischemia Induced TEC Injury

**FIGURE 4:**
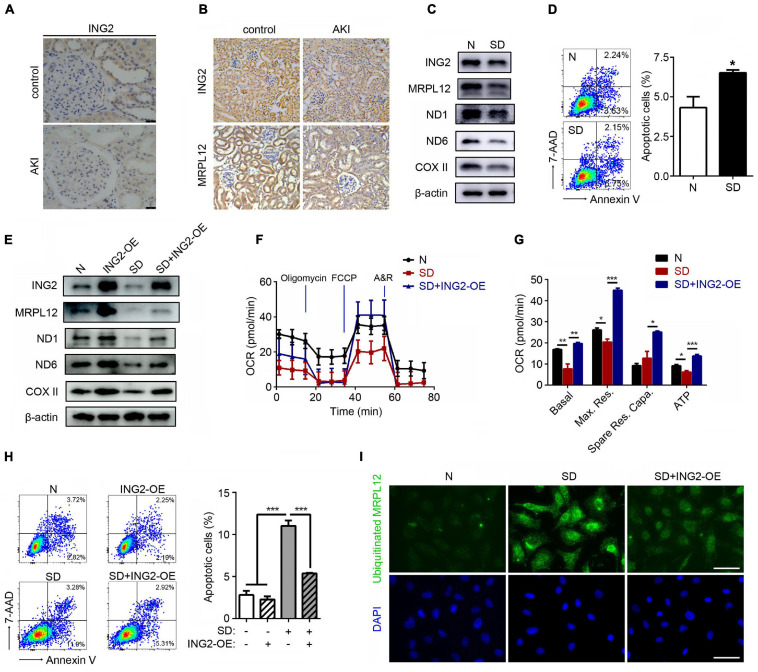
ING2 ameliorated the ischemia induced mitochondrial OXPHOS defects and tubular cell apoptosis. **(A)** Immunostaining of kidney tissue section from healthy individual and AKI patients with antibodies against ING2. At least two patients for each. **(B)** Immunostaining of kidney tissue section from healthy and AKI mice with antibodies against ING2 and MRPL12. HK2 cells were subjected to serum deprivation for 48 h, and protein contents of both ING2, MRPL12, and mtDNA-encoded components of mitochondria complex **(C)** were figured out by Western blotting. The apoptosis of HK2 cells was evaluated by flow cytometry **(D)**. HK2 cells were divided into control group, ING2 overexpression group, serum deprivation group, and serum deprivation plus ING2 overexpression group. MRPL12 and mtDNA-encoded components of mitochondria complex were detected through Western blotting **(E)**. Mitochondrial OXPHOS was detected using Seahorse **(F,G)**. The apoptosis of HK2 cells was evaluated by flow cytometry **(H)**, and the ubiquitination of MRPL12 was determined by proximity ligation assay (PLA) **(I)**. OCR, oxygen consumption rate; FCCP, carbonyl cyanide 4-(trifluoromethoxy)phenylhydrazone; A&R, antimycin and rotenone. Data were from three individual experiments and presented as mean ± SEM. *p < 0.05, **p < 0.01, ***p < 0.001. ns, not significant.

The results above demonstrated that ING2 could promote mtDNA transcription and mitochondrial OXPHOS in TECs at least partly *via* MRPL12. We then asked whether such mechanism is involved in the pathogenesis of AKI. Interestingly, the protein level of ING2 was significantly reduced, especially in tubulointerstitial area of biopsies from AKI patients (Figure 4A). The effects of AKI on ING2 expression were further evidenced in kidney samples from both ischemic kidney injury mice models (Figure 4B) and *in vitro* serum deprived TECs (Figure 4C). At the same time, protein contents of MRPL12 were also reduced by ischemia both *in vivo* and *in vitro* (Figures 4B,C). Protein expressions of OXPHOS complex subunits and mitochondrial OXPHOS capability also decreased, while apoptotic cell populations significantly increased after serum deprivation (Figures 4C,D). Furthermore, overexpression of ING2 could effectively ameliorate serum deprivation induced reduction of MRPL12 and OXPHOS complexes (Figure 4E), impairment of mitochondrial respiration profile (Figures 4F,G), and elevation of cell apoptosis (Figure 4H). Finally, PLA showed a clear increase of ubiquitinated MRPL12 in cells treated with serum deprivation, which could be dramatically reduced by additional ING2 overexpression (Figure 4I). Collectively, these data suggested that ING2 participates in ischemia induced TEC injury.

### Kidney Specific ING2 Overexpression Effectively Ameliorated Ischemic Kidney Injury

**FIGURE 5:**
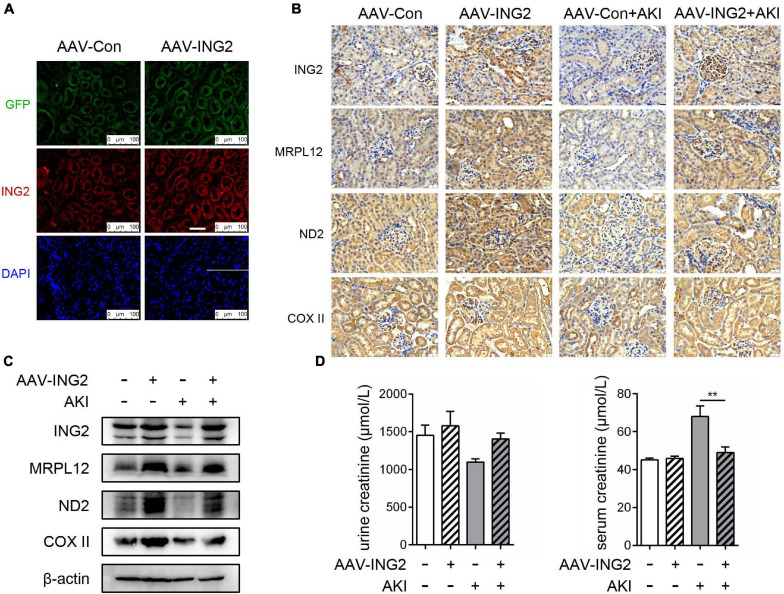
ING2 overexpression effectively ameliorated ischemic kidney injury. The mice were injected with rAAV-con or rAAV-ING2 followed by the induction of IRI with sham operation group as control. **(A)** Immunofluorescent staining of kidneys after the intra-renal injection of rAAV vectors with ING2 (red) and DAPI (blue). **(B)** Immunostaining of renal tissue sections with antibodies against ING2, MRPL12, ND2, and COX II. **(C)** Western blot analysis of renal tissue ING2, MRPL12, ND2, and COX II. **(D)** Levels of creatinine in the serum or urine of IRI and sham operation mice. Data were from two individual experiments and presented as mean ± SEM. rAAV-con + IRI, *n* = 6; rAAV-ING2 + IRI, *n* = 6; rAAV-con + sham, *n* = 4; rAAV-ING2 + sham, *n* = 4. **p < 0.01. ns, not significant.

After the role of ING2, along with its modulating mechanism on MRPL12 and mitochondrial OXPHOS, in ischemic induced TEC injury was established, we continued to determine whether ING2 could serve as one intervening target for ischemic kidney injuries. Results are shown as Figure 5. Immunofluorescent staining revealed the efficiency ING2 overexpression after ING2-AAV transfection (Figure 5A). Consistent with our *in vitro* data, overexpression of ING2 largely ameliorated the effects of acute kidney injury on the levels MRLP12 protein and its downstream targets (Figures 5B,C). At the same time, protein levels of OXPHOS complex ND2 and COX II also increased by ING2 overexpression (Figures 5B,C). Biochemistry analysis revealed significantly decreased serum creatinine levels after ING2 overexpression, indicating ameliorated kidney functions (Figure 5D). These results suggested that ING2 might be an effective target for ischemic kidney injury.

## Discussion

Due to the multiple effects on cell cycle regulation, DNA repair, and senescence, ING2 has gained extensive investigations during the past decades, especially in the field of oncology (Guerillon et al., 2013; Archambeau et al., 2019). Current notion suggested that ING2 might act as one crucial player in the pathogenesis of several tumors, such as non-small cell lung carcinoma, head and neck squamous cell carcinoma, and hepatocellular carcinoma, and serve as a potential pharmacologic target for these cancers as well (Zhang et al., 2008; Borkosky et al., 2009; Ythier et al., 2010; Guerillon et al., 2013). Indeed, experimental studies have demonstrated that ING2 could effectively affect the sensitivity of tumor cells for chemotherapy and radiotherapy and propel tumor cells death (Chin et al., 2005; Zhong et al., 2013). Herein, we defined a novel role of ING2 in maintaining the mitochondrial homeostasis in TECs. By modulating MRPL12 ubiquination, ING2 could regulate the protein contents of MRPL12 and thereby control mtDNA expression, accordingly affect mitochondrial OXPHOS. Furthermore, ING2’s effects on TEC mitochondria are involved in energetic stress induced TEC injuries. As mitochondrial injury has been proposed as one common pathogenic event underlying ample kidney disorders, the identification of ING2 as one modulator of TEC mitochondrial homeostasis might be of significance in exploring potential intervening targets for these diseases.

The results that ING2 induced mitochondrial respiration is accompanied with elevated mRNA levels of OXPHOS subunits propelled us to hypothesize that some transcription regulators of mtDNA might be involved. TFAM is the best elucidated transcription factor for mtDNA. Besides initiating transcription, TFAM is also known to play essential roles in promoting mtDNA replication and maintaining mtDNA stability (Hillen et al., 2017; Ramachandran et al., 2017). While our results that ING2 had no effects on TFAM expression indicated that TFAM might not be involved in ING2-induced mitochondrial respiration (Figures 2A,B). In addition, such findings could also exclude the possibility that p53 participates in ING2’s effects on mtDNA expression. p53 was known to elevate mitochondrial respiration (Liu et al., 2015), and ING2 was evidenced to be capable of enhancing p53 signaling *via* an acetylation modifying manner; however, recent studies revealed that the effects of p53 on mitochondria were mediated mainly by inducing TFAM expression (Saleem and Hood, 2013; Wen et al., 2016).

As the recently identified mtDNA transcription regulator, MRPL12 was significantly increased after ING2 overexpression (Figure 2B), indicating ING2’s effects might be mediated by MRPL12. The subsequent findings that MRPL12 deletion effectively abolished ING2 overexpression induced mtDNA expression and mitochondrial respiration put direct evidences supporting this hypothesis (Figures 2C–E). Different to TFAM, which could induce mtDNA transcription and replication concurrently, it was reported that MRPL12 induced mtDNA transcription was not accompanied by mitochondrial biogenesis (Surovtseva et al., 2011). Our results that ING2 had no effects on mitochondrial copy numbers also coincide with this notion (Figure 1K), which further supports our proposal that ING2 induced mitochondrial respiration was mediated by MRPL12.

The mitochondrial mRNAs are translated into protein by a dedicated set of ribosomes in the mitochondrial matrix. Mitochondrial ribosomes are made up of the 12S and 16S rRNAs encoded by mtDNA and ribosomal proteins imported from the cytoplasm. Our data indicated that ING2 could also modulate 16S rRNA levels (Figure 1H). In addition, MRPL12 acts both as a component of ribosomes and component of mtDNA transcription-related complexes (Wang et al., 2007). All these results raise the possibility that ING2 might also affect mitochondrial function by regulating mitochondrial ribosome biogenesis and subsequent translational activity through MRPL12 and 16S rRNA. Interestingly, ING1b, another member of ING family, can repress rRNA transcription in the nucleolus. ING1b directly binds to nucleolus rDNA, regulates rDNA chromatin modifications, and affects rRNA levels (Rajarajacholan et al., 2017). Although ING2 has exhibited similar functions with ING1 and current works demonstrated that ING2 also localized in mitochondria (Guerillon et al., 2013; Ricordel et al., 2021), it is not clear whether ING2 modulates 16S rRNA transcription by directly binding to mtDNA.

The findings that ING2 induced elevation of MRPL12 protein was not accompanied with concurrent increase of MRPL12 mRNA contents suggested a protein quality and quantity control mechanism might be involved. Ubiquitin proteasome system is the major protein degradation manner for eukaryotic cells, and our bioinformatic analysis suggested that MRPL12 protein did harbor a potential ubiquitinating site (Figure 3B). The following results that ING2 overexpression significantly reduced cellular ubiquitinated MRPL12 contents indicated ING2 could modulate ubiquitin-mediated MRPL12 degradation (Figures 3C,D). To our knowledge, this is the first report concerning the quality control of MRPL12. Besides, such results also coincide the emerging notion that ubiquitin proteasome system participated in mitochondrial biology control (Munch and Harper, 2016; Lavie et al., 2018; Eldeeb et al., 2020). To date, several mitochondrial proteins have been identified as ubiquitinating substance, and proteasome impairments are also evidenced to lead to various mitochondrial injuries (Munch and Harper, 2016; Lavie et al., 2018). However, current notions indicate that there are no proteasomes inside mitochondria. Most of studies investigating the link between the ubiquitin proteasome system and mitochondria focus on ubiquitination of outer mitochondrial membrane (OMM) proteins (Bayne and Trempe, 2019), while a few researches also revealed that intra-mitochondrial proteins, such as Uncoupling proteins (U) and oligomycin sensitivity conferral protein (OSCP), could also be degraded in proteasome-dependent manner (Margineantu et al., 2007; Azzu and Brand, 2010). Pioneer works carried by Lavie et al. demonstrated that the subunit A of succinate dehydrogenase (SDHA), localized in the inner mitochondrial membrane (IMM), can be ubiquitinated in the mitochondria and degraded in proteasome-dependent manner (Lavie et al., 2018). It is noteworthy that MRPL12 is a nuclear DNA encoded protein and should be transported into mitochondrial to exert its biological effects. Whether ubiquitination of MRPL12 occurred within mitochondrial or within cytoplasm and how ubiquitinated MRPL12 was degraded were interesting topics deserving further exploration. Besides, the best elucidated molecular mechanism of ING2 is to modulate acetylation state of its substances such as histone and p53 (Nagashima et al., 2001; Pedeux et al., 2005). Although bioinformatic results suggested MRPL12 also had an acetylating site (Figure 3E), our results demonstrated that ING2 had no effects on MRPL12 acetylation (Figure 3F), thereby indicating MRPL12 might not be the acetylating substance of ING2. Collectively, our results demonstrated that ING2 might exert its modulating effects on mtDNA transcription *via* a ubiquitination mediated MRPL12 degradation manner; however, the mechanism between ING2 and MRPL12 ubiquitination remains to be elusive.

IHC results that ING2 expression was altered in kidney tissue from AKI patients suggested that ING2 might be a pathogenic factor of this kind of kidney disease (Figure 4A). As ischemia is one of the most common environmental attacks of tubular epithelium, we established ischemic circumstance both *in vivo* (ischemic mice) and *in vitro* (serum deprivation stimulation) to test the hypothesis. Both ischemic circumstances led to a significant decrease of ING2 protein levels, along with a concomitant reduction of MRPL12 expression (Figures 4B,C). Furthermore, we demonstrated that ING2 overexpression both *in vitro* and *in vivo* could effectively ameliorate ischemia induced TEC mitochondrial injuries and kidney functions (Figures 4F,G, 5D), providing ING2 as one potential target for ischemic AKI. As mentioned above, mitochondrial dysfunction has been recognized as one common pathogenic event of ample kidney diseases besides AKI, such as renal fibrosis and DKD. Our results might shed new light on exploring future intervenes for these diseases.

It is interesting to note that ING2 expression is often lost or reduced during tumorigenesis as mentioned above, while mitochondrial respiration is also known to be inhibited in several lines of tumors, highlighted by a process termed as Warburg effect (Semenza, 2009; Vander Heiden et al., 2009; Choe et al., 2015). Although Warburg effect has been raised for nearly 100 years, the detailed mechanisms are not fully understood yet. Currently, altered p53 signaling and pyruvate kinase M2 activities have been proposed as possible pathogenic events underlying (Christofk et al., 2008; Eriksson et al., 2017; Liu et al., 2017). Our identification ING2 is a positive regulator for mitochondrial respiration might provide another potential mechanism interpreting Warburg effects, at least in those ING2 lost or reduced tumors, while such possibility deserves further investigation. Interestingly, a recent pioneer work carried by Ricordel et al. demonstrated that ING2 is necessary to maintain mitochondrial ultrastructure integrity without affecting mtDNA transcription in osteosarcoma cell lines (U2OS cells), which seems different to observations (Ricordel et al., 2021). These discrepancy might attribute to the different cell lines used in the experiments. HK2 cell used in our experiments is an immortalized proximal tubule epithelial cell line from normal adult human kidney, which retains relatively normal phenotypic and functional characteristics of human TECs (Ryan et al., 1994). As mentioned above, TECs are one of the most highly energy-demanding cell types within the body, and mainly rely their energy supply on mitochondrial oxidative phosphorylation, while mitochondrial respiration is known to be inhibited in tumors including osteosarcoma cell lines (Giang et al., 2013). Thus, the difference between HK2 cells and osteosarcoma cells in metabolic profiles might contribute to the different effects of ING2 on mtDNA transcription.

In addition, the best elucidated biological function of ING2 is to regulate cell cycle progression, especially G1 to S phase transition either by inducing P21 (Larrieu et al., 2010) or interacting with p53 signaling (Nagashima et al., 2001). Mitochondrial respiration is also known to be adjusted to match the different energy demands at different cell cycle stages. Although the coordination pattern between cell cycle progression and mitochondrial bioenergetics remains large unknown, related studies have identified several cell cycle regulators might play a role, such as cyclin D1-CDK (cyclin dependent kinase) complex, which was evidenced capable of inhibiting Nrf1 signaling, leading to reduced mtDNA expression and subsequently suppressed mitochondrial respiration (Wang et al., 2006). Besides, Cyclin E was also demonstrated to inhibit mitochondrial biogenesis although the detailed intermediate mechanism awaiting exploration (Xu et al., 2014). As ING2 is often reduced during cell mitosis, our findings might provide ING2 as another candidate player during the coordinating process between cell cycle progression and mitochondrial adaption.

In conclusion, our findings showed that ING2 positively modulated mitochondrial respiration in renal TECs and these effects were mediated by MRPL12, mainly through regulating its ubiquitination status. ING2 had a protective role in the ischemia induced impairment of mitochondrial respiration and TEC injury. This finding provided a novel role of ING2 in TEC mitochondrial energetic homeostasis and a potential intervening target for mitochondrial injury associated pathologies.

## Data Availability Statement

The original contributions presented in the study are included in the article/supplementary material, further inquiries can be directed to the corresponding authors.

## Ethics Statement

The animal study was reviewed and approved by Ethics Committee of Shandong Provincial Hospital, Cheeloo College of Medicine, Shandong University.

## Author Contributions

YY and CL designed and performed most of the experiments, interpreted data, and wrote the manuscript. XG, JZ, SZ, and TL assisted in some experiments and data analysis. QW and YL provided the overall guidance and wrote the manuscript. All authors contributed to the article and approved the submitted version.

## Conflict of Interest

The authors declare that the research was conducted in the absence of any commercial or financial relationships that could be construed as a potential conflict of interest.

## Publisher’s Note

All claims expressed in this article are solely those of the authors and do not necessarily represent those of their affiliated organizations, or those of the publisher, the editors and the reviewers. Any product that may be evaluated in this article, or claim that may be made by its manufacturer, is not guaranteed or endorsed by the publisher.
